# Evolution of the bHLH Genes Involved in Stomatal Development: Implications for the Expansion of Developmental Complexity of Stomata in Land Plants

**DOI:** 10.1371/journal.pone.0078997

**Published:** 2013-11-11

**Authors:** Jin-Hua Ran, Ting-Ting Shen, Wen-Juan Liu, Xiao-Quan Wang

**Affiliations:** State Key Laboratory of Systematic and Evolutionary Botany, Institute of Botany, the Chinese Academy of Sciences, Beijing, China; Instituto de Biología Molecular y Celular de Plantas, Spain

## Abstract

Stomata play significant roles in plant evolution. A trio of closely related basic Helix-Loop-Helix (bHLH) subgroup Ia genes, *SPCH*, *MUTE* and *FAMA*, mediate sequential steps of stomatal development, and their functions may be conserved in land plants. However, the evolutionary history of the putative *SPCH/MUTE/FAMA* genes is still greatly controversial, especially the phylogenetic positions of the bHLH Ia members from basal land plants. To better understand the evolutionary pattern and functional diversity of the bHLH genes involved in stomatal development, we made a comprehensive evolutionary analysis of the homologous genes from 54 species representing the major lineages of green plants. The phylogenetic analysis indicated: (1) All bHLH Ia genes from the two basal land plants *Physcomitrella* and *Selaginella* were closely related to the *FAMA* genes of seed plants; and (2) the gymnosperm ‘*SPCH*’ genes were sister to a clade comprising the angiosperm *SPCH* and *MUTE* genes, while the *FAMA* genes of gymnosperms and angiosperms had a sister relationship. The revealed phylogenetic relationships are also supported by the distribution of gene structures and previous functional studies. Therefore, we deduce that the function of *FAMA* might be ancestral in the bHLH Ia subgroup. In addition, the gymnosperm “*SPCH”* genes may represent an ancestral state and have a dual function of *SPCH* and *MUTE*, two genes that could have originated from a duplication event in the common ancestor of angiosperms. Moreover, in angiosperms, *SPCHs* have experienced more duplications and harbor more copies than *MUTEs* and *FAMAs*, which, together with variation of the stomatal development in the entry division, implies that *SPCH* might have contributed greatly to the diversity of stomatal development. Based on the above, we proposed a model for the correlation between the evolution of stomatal development and the genes involved in this developmental process in land plants.

## Introduction

The origin of terrestrial plants was a key event in the evolutionary history of life on earth [1], [2]. During the transition to a terrestrial habitat, ancestral land plants overcame several challenges, including growing in an environment with a limited water and mineral supply, surviving the harmful effects of enhanced ultraviolet and cosmic rays and defending against attack from a new and diversified set of microbes [2]. Stomata were a key evolutionary innovation that contributed to overcoming many of these challenges. Stomata first appeared more than 410 million years ago [3], [4] and generally consist of two guard cells (GCs) surrounding a pore in the epidermis except that a single guard cell that encircles the pore was found in the moss *Funaria* [5]. They play significant roles in protecting plants from a non-aqueous atmosphere by mediating gas exchange integral to photosynthesis and water transportation [4], [6]. Some of the earliest branching land plants, including liverworts, hornworts and some mosses, lack stomata, but all other land plants possess them [3]. Fossil evidence shows that the structure of stomata has remained more or less unchanged ever since its origin [3], [7], [8], but the developmental processes that lead to stomata have become more complex, such as the occurrence of amplifying divisions, and also more efficient throughout the land plant phylogeny [4], [9], [10]. These efficiencies in water use, gas exchange and photosynthetic rates are likely key to driving the adaptation of higher plants to a wide array of ecological niches although little is known about possible correlations between stomatal patterning and physiological function [9]–[19].

To date, most investigations of stomata have focused on their development and their reactions to environmental stressors (e.g., [3], [9], [13], [20]–[23]). However, in the last decade some of the genes that regulate stomatal development and patterning have been isolated. In *Arabidopsis*, these genes are a trio of closely related basic Helix-Loop-Helix (bHLH) genes, known as *SPCH* (*SPEECHLESS*), *MUTE*, and *FAMA* (here termed the *SMF* genes). These three genes work together with their heterodimeric partners - *SCRM* (*SCREAM*) and *SCRM2* - to mediate sequential steps of the cell-state transitions that lead to stomatal formation. These steps include: *i*) asymmetric cell division that leads to *ii*) the acquisition of guard mother cell (GMC) identity and, finally, *iii*) the differentiation of guard cells (GCs) [24]–[28].

The bHLH family, which is typified by the highly conserved bHLH domain, is a large family of transcription factors that is distributed throughout the major eukaryotic lineages [29]. Phylogenetic analysis indicates that the bHLH genes from *Arabidopsis* are divided into 26 subgroups [27], [29], [30]. The three genes that contribute to stomatal development - i.e., *SPCH*, *MUTE*, and *FAMA* - belong to bHLH subgroup Ia, which also includes seven genes of unknown function. In contrast, *SCRM* and *SCRM2* are members of subgroup IIIb [29].

The function of Ia and IIIb genes may be conserved over evolutionary time. For example, functional analyses have shown that PpSMF1 and PpSMF2, two bHLH group Ia members from the bryophyte *Physcomitrella patens*, can recapitulate elements of the *SPCH*, *MUTE* and *FAMA* overexpression phenotypes of *Arabidopsis thaliana* [31]. PpSMF1 can also partially complement *mute* and *fama* mutants in *A. thaliana* [31]. In addition, the partnership between the subgroup Ia SPCH/MUTE/FAMA and the subgroup IIIb SCRM/SCRM2 might have an ancient origin, because both of these two subgroups occur in the basal land plant, *P. patens* [32].

The evolutionary history of *SPCH/MUTE/FAMA* genes is nonetheless controversial [31], [33] because there is conflicting information about their origins and the relationships of their paralogs. At least two analyses found that the *SPCH* and *MUTE* genes are closest paralogs [29], [34], [35], whereas others suggest that *SPCH* and *FAMA* genes are closest paralogs [31], [33]. There are also discrepancies as to whether the homologs from basal land plants fall within the *SPCH*, *MUTE* and *FAMA* clades or represent sister-lineages to these clades [31]. It is important to note that the resolution of these phylogenetic issues will yield insight into the evolution of stomata, because each of the bHLH Ia genes performs a defined role in stomatal development.

Here we investigate the distribution and evolutionary relationships of bHLH Ia genes among land plants to better understand the evolution of genes involved in stomatal development. Thus far, evolutionary analyses of the members of Ia subgroup have been based on a small sample (n <12) of angiosperm species; here we survey a total of 51 species of land plants (and one green algae and two multicellular algae species) for the presence and distribution of subgroup Ia genes. Based on both phylogenetic and structural analyses of the bHLH Ia genes, we interpret their evolution in land plants, and we also predict a model for the correlation between the evolution of stomatal development and the genes involved in their development.

## Materials and Methods

### Ethics Statement

No specific permits were required for the sampling.

### Identification of bHLH Ia Homologs

We surveyed a number of plant databases – such as Phytozome, NCBI, PGDD, PlantTFDB, EST, SRA and FLcDNA databases and other genome databases (Tables 1, 2, S1) - to identify bHLH Ia homologs from plant species. To retrieve Ia homologs from these databases, we performed tBLASTn and BLAST+ (ncbi-blast-2.2.27+) searches using the *A. thaliana FAMA* [Phytozome:AT3G24140] amino acid sequence as a query.

**Table 1:** Information of the bHLH Ia genes in the sampled plant species with whole genome sequences.

Species	Abbr.	Copy number				Database*
		*FAMA*	*MUTE*	*SPCH*	Other Ias	
*Volvox carteri*	Vca	0	0	0	0	Phytozome
*Physcomitrella patens*	Ppa	2	0	0	0	Phytozome/PGDD
*Selaginella moellendorffii*	Smo	3	0	0	0	Phytozome/PGDD
*Amborella trichopoda*	Atr	1	1	1	2	Amborella GD
*Aquilegia coerulea*	Aco	1	1	1	2	Phytozome
*Arabidopsis lyrata*	Aly	1	1	1	7	Phytozome/PGDD
*Arabidopsis thaliana*	Ath	1	1	1	7	Phytozome/PGDD
*Brachypodium distachyon*	Bdi	1	1	2	7	Phytozome/PGDD
*Brassica rapa*	Bra	3	3	3	14	Phytozome/PGDD
*Cajanus cajan*	Cca	1	1	1	7	IIPG/PGDD
*Capsella rubella*	Cru	1	1	1	9	Phytozome
*Carica papaya*	Cpa	1	0	1	4	Phytozome/PGDD
*Citrus clementina*	Ccl	1	1	1	3	Phytozome
*Citrus sinensis*	Csi	1	0	1	4	Phytozome
*Cucumis sativus*	Csa	1	1	1	5	Phytozome/PGDD
*Eucalyptus grandis*	Egr	1	1	1	5	Phytozome
*Fragaria vesca*	Fve	0	1	0	6	PFR/PGDD
*Glycine max*	Gma	2	2	4	12	Phytozome/PGDD
*Linum usitatissimum*	Lus	1	1	4	7	Phytozome
*Lotus japonicus*	Lja	0	1	0	6	Kazusa/PGDD
*Malus domestica*	Mdo	1	2	2	8	Phytozome/PGDD
*Manihot esculenta*	Mes	2	2	2	7	Phytozome
*Medicago truncatula*	Mtr	0	0	1	5	Phytozome/PGDD
*Mimulus guttatus*	Mgu	0	0	2	2	Phytozome
*Musa acuminata*	Mac	2	2	3	16	Banana Genome/PGDD
*Oryza sativa*	Osa	1	1	2	8	Phytozome/PGDD
*Phaseolus vulgaris*	Pvu	1	1	2	6	Phytozome
*Phoenix dactylifera*	Pda	1	2	1	5	Date Palm Draft Sequence
*Populus trichocarpa*	Ptr	2	1	2	9	Phytozome/PGDD
*Prunus persica*	Per	1	1	1	5	Phytozome/PGDD
*Ricinus communis*	Rco	1	1	1	4	Phytozome/PGDD
*Setaria italica*	Sit	1	1	2	8	Phytozome
*Solanum lycopersicum*	Sly	2	1	0	4	SGN/PGDD
*Solanum tuberosum*	Stu	2	0	0	4	PGSC/PGDD
*Sorghum bicolor*	Sbi	1	1	2	7	Phytozome/PGDD
*Thellungiella halophila*	Tha	2	1	1	8	Phytozome
*Theobroma cacao*	Tca	1	1	1	5	CIRAD/PGDD
*Vitis vinifera*	Vvi	1	1	1	5	Phytozome/PGDD
*Zea mays*	Zma	1	1	3	10	Phytozome/PGDD

Abbr., Abbreviation.*, database websites: Amborella GD, http://www.amborella.org/; Banana Genome, http://banana-genome.cirad.fr/; CIRAD, http://cocoagendb.cirad.fr/gbrowse/download.html; Date Palm Draft Sequence, http://qatar-weill.cornell.edu/research/datepalmGenome/download.html; IIPG, http://www.icrisat.org/gt-bt/iipg/Home.html; Kazusa, ftp://ftp.kazusa.or.jp/pub/lotus/; NCBI, http://www.ncbi.nlm.nih.gov/; Phytozome, http://www.phytozome.net; PFR, http://www.strawberrygenome.org/; PGSC, http://potatogenomics.plantbiology.msu.edu; PlantTFDB, http://planttfdb.cbi.edu.cn/.

**Table 2:** Information of the bHLH Ia genes in the plant species with EST or SRA databases.

	Species	Copy Number				EST & cDNA	SRA (spots)	SRA Submission
		*SPCH*	*MUTE*	*FAMA*	Other Ias			
**Green algae**	*Nitella hyalina*	–	–	–	–	88,280	949,065	SRA023590
	*Nitella mirabilis*	–	–	–	–	83,526	–	–
**Liverwort**	*Marchantia polymorpha*	–	–	–	–	33,722	22,854,396	SRA026315
**Fern**	*Adiantum capillus-veneris*	–	–	–	–	30,544	–	–
**Gymnosperms**	*Cephalotaxus harringtonia*	–	–	–	1	–	695,559	SRA023613
	*Ginkgo biloba*	–	–	1	3	21,709	64,057	SRA030487
	*Gnetum gnemon*	–	–	–	1	10,756	432,517	SRA023615
	*Picea glauca#*	1	–	–	1	321,713	10,922,903	SRA023921
	Picea breweriana*	1	–	1	–	–	–	–
	Picea jezoensis*	1	–	1	–	–	–	–
	Picea smithiana*	1	–	1	–	–	–	–
	*Picea sitchensis*	–	–	–	1	206,402	–	–
	*Pinus banksiana*	–	–	1	–	36,387	1,397,993	SRA048732
	*Pinus taeda*	–	–	1	7	329,066	4,331,325	SRA023533
	*Sciadopitys verticilliata*	–	–	–	1	–	484,806	SRA023758

#, Sequences from FLcDNA database of Arborea; *, sequences from PCR amplification; -, missing information.

We subjected the identified sequences to three additional filters. First, we discarded sequences that had lower sequence similarities to AT3G24140 than did the first non-subgroup-Ia member from *A. thaliana* [Phytozome: AT2G22750, a member of IVa]. Second, we examined individual sequences for two characteristic features of subgroup Ia genes: the bHLH domain and the SMF domain [30], [31]. These two domains were identified by Heim *et al.* [30]. Any sequence that lacked one of the domains was removed from further analysis. In addition, 36 putative Ia genes were modified by hand for a better alignment with the bHLH or SMF domain (Table S1). Finally, we culled redundant transcripts, sequences with incomplete bHLH or sequences with unalignable C-terminal domains from the final dataset.

### DNA and RNA Extraction, PCR and RT-PCR Amplification, Cloning and Sequencing

Two types of bHLH Ia genes that grouped with *SPCH/MUTE/FAMA* genes were found in eight gymnosperm species, but we found no species that had both types. To assess whether these two types existed in one species, we amplified these Ia genes from the DNA and cDNA of three *Picea* species (*Picea breweriana*, *P. jezoensis*, and *P. smithiana*). Genomic DNAs were extracted from leaves using the modified cetyltrimethylammonium bromide (CTAB) method [36], [37]. Total RNA extraction, purification and first-strand cDNA synthesis followed the protocols of Guo *et al*. [38]. The primers used to amplify the Ia genes were designed to the EST sequence [GenBank:GW768467] of *Pinus banksiana* and the predicted unigene [Arborea:GQ04006] from *Picea glauca*, including FAMA-U5F3 and FAMA-U3R as well as bHLH2-U5F and bHLH2-U3R1 (Table S2). Polymerase chain reaction (PCR), product purification, cloning and sequencing followed the protocols of Ran *et al*. [39] except an annealing and sequencing reaction temperature of 60°C. In addition, some internal primers were used in sequencing (Table S2).

### Phylogenetic Reconstruction

Coding sequences of bHLH Ia genes were aligned using the program Clustal X version 2.0 [40]. The conserved bHLH and SMF domains (a total of 468 bp or 156 aa) [31] were used for the phylogenetic analyses. Substitution saturation was tested using DAMBE 5.3.00 [41], which showed that the third positions were saturated. jModeltest 2 [42] and ProtTest 3.2 [43] were used to identify the best-fit models of nucleotide and amino acid (AA) sequence evolution, respectively. Using both Akaike Information Criterion (AIC) and Bayesian Information Criterion (BIC), the GTR+I+G model was selected for nucleotide sequence analyses and the JTT+I+G model for AA analyses. Bayesian and maximum-likelihood (ML) trees were constructed with MrBayes version 3.1.2 [44] and PhyML version 2.4.4 [45], respectively.

### Selection Test and Nucleotide Diversity Analysis

**Figure 1:**
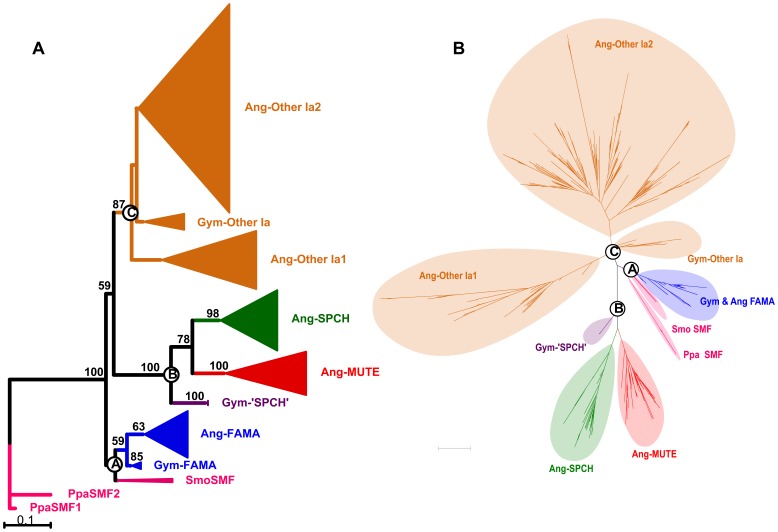
Maximum likelihood (ML) trees of the bHLH Ia genes constructed based on the nucleotide sequences. A. The tree was rooted with the Ia genes from *Physcomitrella patens*; B. The tree was not rooted. Numbers above branches indicate bootstrap values higher than 50%. Ang, angiosperm; Gym, gymnosperm; Smo, *Selaginella moellendorffii*; Ppa, *Physcomitrella patens*.

ML analyses were performed to identify regions or clades of the bHLH Ia genes that may have been subject to diversifying selection, using the Fitmodel program version 0.5.3 [46] and the codeml program of PAML version 4.6 [47], respectively. Because the Ia gene dataset was very large, we limited these analyses to a subset of subgroup Ia sequences. *SPCH/MUTE/FAMA* homologs, all bHLH other Ias from gymnosperms, all bHLH Ias from *Physcomitrella* and *Selaginella*, and some of bHLH other Ias from four angiosperm species (*Arabidopsis thaliana*, *Glycine max*, *Linum usitatissimum* and *Oryza sativa*) were analyzed with Fitmodel, in which the M0, M3, M3+S1 and M3+S2 models were applied. Three genic classes, including clade A, and Ang-MUTE and Ang-SPCH in clade B (Fig. 1A), were analyzed with the branch-site and site models (codeml program), respectively. The parameter settings followed Guo *et al*. [38]. To test the evolutionary rate variation, DnaSP version 5.10.00 [48] was used to calculate the nucleotide diversity (*Pi*), the number of nonsynonmous substitutions per nonsynonymous site (*Ka*), the number of synonymous substitutions per synonymous site (*Ks*), and the ω (*Ka*/*Ks*) value for the sequences of the angiosperm *FAMA*, *MUTE*, *SPCH* and other Ia genes (Fig. 1) because some clades lack members from non-flowering plants.

## Results

### Sequence Characterization

We searched complete genomes as well as EST and SRA resources to identify bHLH Ia homologs in basal (non-angiosperm) land plants. Using Blast-based strategies (see Materials and Methods) with a loose searching criteria (E value = 1), we characterized two and three SMF genes in the bryophyte *Physcomitrella patens* and the lycopsid *Selaginella moellendorffii*, respectively. However, using similar search methods, we were unable to identify bHLH Ia homologs from the complete genome of a green algae (*Volvox carteri*), and the EST+cDNA databases of two multicellular green alga (*Nitella hyalina* and *Nitella mirabilis*) (88,280 and 88,526 sequences, respectively), a liverwort (*Marchantia polymorpha*) (33722 sequences) and a fern *Adiatum capillus-veneris* (30,544 sequences) (Table 2). The inability to identify Ia homologs from EST databases of the liverwort and fern could reflect reality, or it could be caused by the size of EST databases. We note, however, that other non-Ia bHLH genes were successfully retrieved from these species. In addition, we identified 19 Ia homologs from the EST or SRA databases of eight gymnosperms (Table S1), and PCR-amplified six sequences from three spruce species (Table 2). Of the 25 sequences from gymnosperms, 4, 6 and 15 showed similar gene structures with *SPCH*, *FAMA* and Other Ias, respectively.

Within angiosperms, SMF (*SPCH*, *MUTE* and *FAMA*) homologs were identified in all 36 species sampled, but homologs of individual genes were not found in five species for *FAMA*, four species for *SPCH* and five species for *MUTE*. However, 14, 6, and 8 species harbored more than one *SPCH*, *MUTE* and *FAMA* homolog (Table 1). In addition, 2–16 copies of other (i.e., non-SPCH, non-MUTE and non-FAMA) Ia members were found throughout the 36 angiosperm species. In total, 395 bHLH Ia sequences from 49 species of land plants were used in the final analyses (Table S1). These coding sequences ranged from 543 (AcoMUTE) to 3114 bp (FveIa_1).

**Figure 2:**
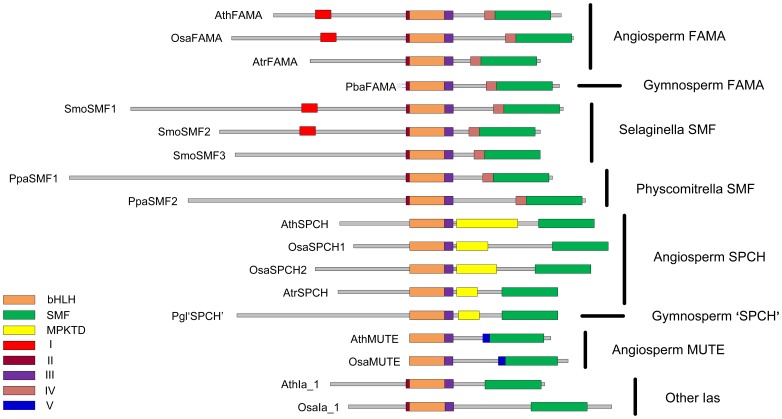
Structural diagram of the bHLH Ia genes from some representative species of land plants. The regions that are unique to *FAMA*, *MUTE*, and *SPCH* are marked with different colors.

All of the sequences in the final dataset contained both bHLH and SMF domains, but additional domains were also detected (Fig. 2), as suggested by MacAlister and Bergmann [31]. These domains helped to differentiate gene structure among putative *SPCH*, *MUTE* and *FAMA* genes and also to corroborate phylogenetic inferences based on the bHLH and SMF domain sequences (see below). In angiosperms, the *FAMA* homologs have a pre-bHLH domain (II) and two unique and highly conserved regions; one of these (I) is in the N-terminus between the start codon and the bHLH domain and the other (IV) is immediately N-terminal to the SMF domain. However, four putative *FAMA* homologs (AtrFAMA CpaFAMA, EgrFAMA, and StuFAMA) lacked region I. All *MUTE* homologs lacked the pre-bHLH domain (II) and had a unique pre-SMF domain (V). Finally, all *SPCH* homologs had a MPKTD domain, but it differed in length.

**Figure 3:**
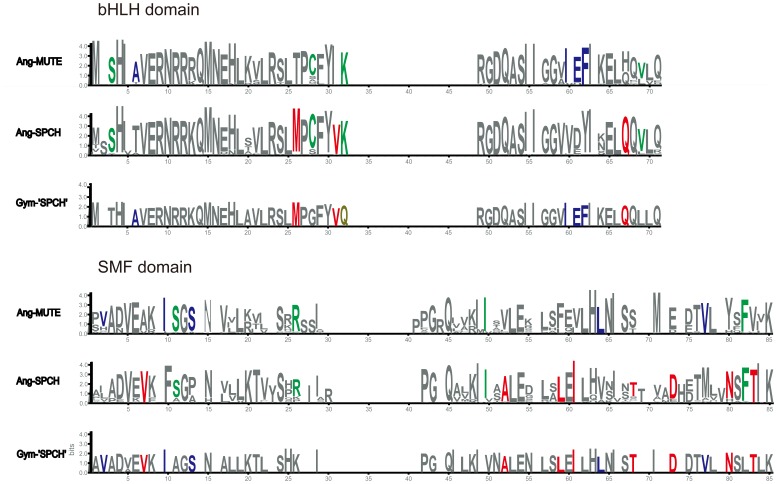
Graphical sequence logo representation of bHLH and SMF domains of seed-plant *SPCH/MUTE* genes. The conserved sequence pattern was generated using WebLogo3 (http://weblogo.berkeley.edu/). Bits represent the relative frequency of amino acids.

The gene structures in non-angiosperm taxa merit special attention. In gymnosperms, two structures were found, one similar to angiosperm *FAMA* genes and the other similar to angiosperm *SPCH* genes. In *Selaginella moellendorffii*, the three bHLH Ia genes are structurally similar to the angiosperm *FAMA* genes, especially in domains II and IV, and the domain I was also found in SmoSMF1 and SmoSMF2. In *Physcomitrella patens*, the two Ia genes are also similar to the angiosperm *FAMA* genes due to the presence of domains II and IV. Moreover, the other Ia genes also have domain II (Fig. 2). In brief, according to the gene structure analysis, the basal land plants (bryophyte and lycopsid) only harbored *FAMA* Ia genes, while both *FAMA* and *SPCH* Ia genes occurred in gymnosperms. This was consistent with the reconstruction of ancestral gene structure using Mesquite version 2.75 [49], which is shown in Fig. S1. The conserved sequence patterns of the angiosperm *SPCH* and *MUTE* genes as well as the gymnosperm ‘*SPCH*’ genes were generated with WebLogo3 (http://weblogo.berkeley.edu/) and shown in Fig. 3.

### Phylogenetic Analyses

The ML trees generated by nucleotide and AA sequences of the bHLH Ia genes are topologically identical except some branches with low bootstrap support (Figs. 1, S2). Because most Bayesian prosterior probabilities were lower than 0.90, we did not consider the Bayesian tree. The ML trees of the nucleotide sequences with and without outgroups are shown in Figure 1A and 1B, respectively. From the rooted trees, we found that the Ia genes of vascular plants are monophyletic with high bootstrap support (100%) and could be divided into three main clades (A, B and C) (Figs. 1A, S2A, S2B). Clade A included the SMF genes from *Selaginella moellendorffii* and the *FAMA* homologs from seed plants (gymnosperm and angiosperm). Clade B comprised two sister subclades, i.e., the gymnosperm ‘*SPCH*’ genes and the angiosperm *MUTE* and *SPCH* genes. Clade C comprised all the other Ia genes from gymnosperms and angiosperms. From the unrooted tree, the bHLH Ia members from *Physcomitrella patens* grouped with Clade A (Figs. 1B, S2C).

### Selection Test and Nucleotide Diversity

The likelihood ratio test (LRT) of the nested models in the Fitmodel program showed that the M3+S1 model was significantly better than the other ones. In the M3+S1 model, the switching rates between ω values (ω1 to ω2, ω1 to ω3, and ω2 to ω3) are equally imposed. Under this model, no codon or branch was inferred to be under relaxed selection (ω3 = 0.32) (Table 3).

**Table 3:** Results of the LRT test of the models in Fitmodel for the bHLH Ia genes.

	M0	M3	M3+S1	M3+S2
***lnL***	−26059.06	−25848.39	−25656.58	−25707.45
**ω1 ω2 ω3**	0.08	0.01 0.07 0.20	0.00 0.07 0.32	0.01 0.19 58.10
***p*** **1 ** ***p*** **2 ** ***p*** **3**	1.00	0.31 0.51 0.17	0.40 0.42 0.18	0.65 0.34 0.00
***R*** **01 ** ***R*** **02 ** ***R*** **12**			1.59 1.59 1.59	1.66 9.02 434.75

The site model test indicated that no site in the three classes A, Ang-MUTE and Ang-SPCH (Fig. 1) experienced positive selection. Using the branch-site model, no positively selected site was found in the angiosperm *SPCH* or *MUTE* genes when they acted as the background clade to each other. For the *FAMA* homologs, four branches (vascular plants, seed plants, angiosperms, and eudicots) were tested, and one positively selected site was detected in the branches of seed plants and angiosperms, respectively, but the positive selection was not supported by the LRT test due to a significance much higher than 0.05 (Table S3).

For angiosperms, the *FAMA* genes showed the lowest *Pi*, *Ka* and *ω* values, while the *SPCH* and *MUTE* genes had similar *Pi*, *Ka*, *Ks* and *ω* values. In addition, the highest *Pi*, *Ka*, *Ks* and *ω* values were found in the other Ia genes (Table 4).

**Table 4:** Sequence information of angiosperm *SPCH*, *MUTE*, *FAMA* and Other Ias.

Dataset	*N*	*Pi*	*Ka*	*Ks*	*ω* (*Ka/Ks*)
*SPCH*	53	0.25742±0.00454	0.11749	0.69853	0.168
*MUTE*	38	0.25526±0.00887	0.11350	0.73050	0.155
*FAMA*	41	0.21573±0.00868	0.05844	0.72591	0.091
Other Ias	233	0.29867±0.00363	0.16091	0.75129	0.214

*N*, Number of sequences; *Pi*, Nucleotide diversity; *Ka*, The number of nonsynonymous substitutions per nonsynonymous site; *Ks*: The number of synonymous substitutions per synonymous site; ω: *Ka*/*Ks*.

## Discussion

### Evolutionary History of the bHLH Ia Genes Involved in Stomatal Development

Genome-wide analyses of the bHLH transcription factor family have found that SPCH, MUTE and FAMA, together with some other proteins of unknown functions, form a well supported clade (subgroup Ia) [27], [29], [30], [35], which is characterized by a conserved region of 18 amino acids immediately N-terminal to the bHLH domain and a domain at the C-terminus of the protein [30], [31]. Some previous phylogenetic analyses found that the *SPCH* and *MUTE* genes were the closest sister paralogs (The *SPCH* genes were not monophyletic) and the *FAMA* genes grouped with some other subgroup Ia members [29], [34], [35]. However, other studies that focused on the SMF (*SPCH*, *MUTE*, *FAMA*) genes did not obtain the same phylogenetic relationships. For example, Liu *et al*. [50] found that the *SPCH/MUTE/FAMA* genes from *Arabidopsis thaliana*, *Oryza sativa* and *Zea mays* formed three monophyletic groups, respectively, although the functions of SPCH and MUTE from *Oryza* are somewhat divergent from their homologs in *Arabidopsis*. In contrast, by sampling *Arabidopsis*, *Populus*, *Oryza*, and *Physcomitrella*, Peterson *et al*. [33] found that the angiosperm *SPCH* and *MUTE* genes formed a clade sister to a clade comprising the angiosperm *FAMA* genes and the *Physcomitrella* bHLH Ia members. In addition, MacAlister and Bergmann [31] constructed a phylogeny of the SMF genes based on a sampling of nine angiosperms plus *Selaginella moellendorffii* and *Physcomitrella patens* with complete genome sequences and a EST sequence (*Pgl*‘*SPCH’* [*PgSPCH*]) from the conifer *Picea glauca*. They found that the angiosperm *SPCH*, *MUTE* and *FAMA* genes formed monophyletic groups, respectively, and *SPCH* was closer to *FAMA* than *MUTE*. Particularly, *SmoSMF2* (*SmSMF2*) and *SmoSMF3* (*SmSMF3*) (Ia members of *Selaginella*) were sister to the angiosperm *FAMA* genes, while *SmoSMF1* (*SmSMF1*) (also an Ia member of *Selaginella*), *PpaSMF1* (*PpSMF2*) and *PpaSMF2* (*PpSMF2*) (Ia members of *Physcomitrella*) were sister to the angiosperm *MUTE* genes with strong bootstrap supports, and *PglSPCH* was sister to the angiosperm *MUTE* genes with a weak support (49%). This phylogeny also seems to be supported by the domain architectures of the genes that they designated [31]. Therefore, as discussed above, the evolutionary history and patterns of the bHLH Ia genes involved in stomatal development are still greatly controversial, especially the phylogenetic positions of the Ia genes from basal land plants and gymnosperms.

In the present study, putative homologs of the bHLH Ia genes were identified from 49 species representing four major lineages of land plants (bryophyte, lycophyte, gymnosperm, and angiosperm). Because previous phylogenetic studies indicate that the bHLH Ia genes are monophyletic [26], [29], [34], [35] and non-bHLH amino acid motifs are highly conserved in each bHLH subgroup [29], [30], we only include the bHLH Ia members in this study in order to avoid incorrect phylogenetic topology that could be caused by improper alignment between different subgroups. Also, considering that the Ia genes first appeared in *Physcomitrella patens*, the two Ia members from this species (*PpaSMF1/2*) were used as functional outgroups in the phylogenetic analysis. The ML trees based respectively on the amino acid sequences and the first plus second codon positions both support that the Ia genes from vascular plants could be divided into three clades. That is, Clade A comprises the SMF genes from *Selaginella moellendorffii* and the *FAMA* homologs from seed plants (gymnosperm and angiosperm); Clade B includes two monophyletic sister groups, i.e., the gymnosperm “*SPCH*” genes and the angiosperm *MUTE* and *SPCH* genes; and Clade C includes other Ia members from gymnosperms and angiosperms that could not be grouped with *SPCH/MUTE/FAMA* (Fig. 1A). The unrooted ML tree also supports the three clades and a close relationship between the Ia genes from *Physcomitrella patens* and Clade A (Fig. 1B).

The *SPCH*, *MUTE* and *FAMA* genes of angiosperms form monophyletic groups, respectively, which is corroborated by their different gene structures (Figs. 1, 2). All *SPCH* genes have the unique and conserved MPKTD domain, although with different lengths, and this domain is important for regulating the SPCH activity in response to phosphorylation by the MAP kinases [51]. In addition, except *PvuSPCH1* and *LusSPCH3*, all other SPCHs have a conserved position of stop codon. The *MUTE* genes have a unique conserved region (V) [31], and lack some residues preceding the bHLH domain that are present in all the other bHLH Ia members with various lengths. In contrast, the *FAMA* genes have high AA sequence similarity (Table 4), and harbor three unique domains (I, II and IV) (Fig. 2).

For gymnosperms, the *FAMA* and ‘*SPCH’* genes were found in the EST databases of *Ginkgo biloba*, *Picea glauca*, *Pinus banksiana* and *Pinus taeda* (Tables 2, S1), and were successfully PCR-amplified from *Picea breweriana*, *P. jezoensis* and *P. sitchensis*. We also tried to amplify the ‘*MUTE’* genes from gymnosperms using different primers, but failed. Moreover, we blasted the draft genome sequence of *Picea abies* just released, which could have covered 96% of the protein-coding genes of this species [52], and found four bHLH Ia genes [ConGenIE:MA_57244g0010, ConGenIE:MA_686524g0010, ConGenIE:MA_120602g0010 and ConGenIE:MA_130776g0010] (http://congenie.org). When the four genes were added into our dataset, the generated tree is topologically identical to Figure 1 and indicates that MA_57244g0010 is a *FAMA* homolog and MA_120602g0010 is a ‘*SPCH’* homolog. The other two genes belong to other Ias (Tree not shown). Therefore, all available evidence strongly suggests that the *MUTE* gene does not occur in gymnosperms.

Undoubtedly, as suggested by MacAlister and Bergmann [31], the gymnosperm *FAMA* genes are sister to the angiosperm *FAMA* genes (Fig. 1), and the sister relationship is also supported by the high similarities of gene structures (Fig. 2). However, the gymnosperm ‘*SPCH’* genes are sister to a clade comprising the angiosperm *SPCH* and *MUTE* genes with 100% bootstrap support, although they are very similar to the angiosperm *SPCH* genes in structure, especially the presence of the N-terminal sequence before the bHLH domain and the partial MPKTD domain (Fig. 2). By comparing the AA sequences of the bHLH and SMF domains, we found that the gymnosperm ‘*SPCH’* genes are similar to the angiosperm *SPCH* sequences in some sites, but are identical to the angiosperm *MUTE* sequences in some other sites (Fig. 3). Additionally, deletion of the MPKTD domain of SPCH can generate proteins with functions similar to MUTE [27], [51]. Therefore, we deduce that the gymnosperm ‘*SPCH’* genes may be closely related to and function as the common ancestor of the angiosperm *SPCH/MUTE* genes, and the *MUTE* genes could be derived from the ancestral copy by losing the MPKTD domain and the N-terminal sequences before the bHLH domain. This inference is also consistent with the reconstruction of ancestral gene structure (Fig. S1).

All bHLH Ia genes from *S. moellendorffii* are located in Clade A (FAMA), which is in line with Pires and Dolan [29] and Peterson *et al*. [33] but different from MacAlister and Bergmann [31]. In MacAlister and Bergmann [31], SmoSMF1 was placed sister to the angiosperm *MUTE* genes whereas SmoSMF2–3 were sister to the angiosperm *FAMA* genes. Comparing sequences of the conserved domains and structures of these genes, we found that all members from *Selaginella* have the pre-SMF domain (IV) and the unique region prior to the bHLH domain (II) (Fig. 2). Meanwhile, SmoSMF1 and SmoSMF2 have the conserved region in the N-terminus (I) that occurs in most angiosperm *FAMA* genes. This information, together with the high AA sequence similarity among *SmoSMF*s and the *FAMA* genes of gymnosperms and angiosperms (data not shown) and the phylogenetic reconstruction (Fig. 1), strongly suggests that all bHLH Ia members of *Selaginella* have close relationships with the *FAMA* genes. In addition, the function of the *FAMA* genes might be ancestral in bHLH Ia subgroup because *PpaSMFs* and *SmoSMFs* from the basal land plants are structurally identical and closely related to the *FAMA* genes of gymnosperms and angiosperms (Figs. 1, 2, S1).

Among the bHLH Ia genes from angiosperms, the *FAMA* genes are more conserved than the other ones, showing not only the lowest nucleotide diversity (*Pi* = 0.21573), but also the lowest nonsynonymous substitution rate (*Ka* = 0.05844) and *Ka/Ks* ratio (*ω* = 0.091). The *SPCH* and *MUTE* genes have similar values of nucleotide diversity, nonsynonymous and synonymous substitution rates, and *Ka/Ks* (Table 4). Therefore, the *SPCH/MUTE/FAMA* genes might have experienced different selective pressures, although the fact that no site or branch was detected to be under positive or relaxed selection using Fitmodel and codeml suggests conserved functions of all of them (Table 3, Table S3). Nevertheless, the functional shift could have occurred repeatedly in the *SPCH* genes, considering that more copies of them are maintained in some angiosperm species (Table 1), and that the functional divergence of different *SPCH* copies has been reported in *Oryza sativa* and *Zea mays* [50]. It cannot be ruled out that the multiple conspecific copies of the bHLH Ia subgroup, especially for the *SPCH/MUTE/FAMA* genes, were caused by ancient or recent genome duplications (http://chibba.agtec.uga.edu/duplication/). For example, some species that have experienced recent whole-genome duplication (WGD), such as *Brassica rapa*, *Glycine max*, *Musa acuminate*, *Populus trichocarpa*, *Solanum lycopersicum*, *Solanum tuberosum* and *Zea mays*, harbor more bHLH Ia genes. However, recent WGD cannot explain why more *SPCH* genes are retained than the *MUTE* and *FAMA* genes in some species (Table 1). Until now, no functional studies have been performed for other bHLH Ia genes (Fig. 1A, Clade C). These genes experienced frequent duplication and extinction events, both ancient and recent, and thus it would be interesting to investigate their functional differentiation in the future (Fig. S2).

### Evolution of Stomatal Development in Land Plants

The developmental process of stomata has evolved and become complex with the evolution of land plants, especially the occurrence of amplifying divisions [14]. This process has been well studied in *Arabidopsis*, which includes three main steps, i.e., asymmetric entry divisions of the meristemoid mother cells (MMCs) to create meristemoids (M), self-renew via amplifying divisions or direct differentiation of meristemoids into GMCs, and symmetrical divisions of GMCs to form GCs [53]. However, in mosses and lycophytes, stomata develop by a simple process, in which a single asymmetric or symmetric division is followed by differentiation of a GMC, then GCs ( [54],reviewed by [55]). For ferns, an epidermal cell may go through one or two asymmetric divisions, then differentiates into a GMC, and finally into GCs [56]. In gymnosperms, different stomatal development patterns have been reported. The meristemoid divides once symmetrically to generate a GMC that then develops into GCs in *Pinus* [57], but it is interesting that *Ginkgo biloba*, a living fossil of gymnosperms, possesses both asymmetric divisions in perigenous neighbor cells like grasses and amplifying divisions within the stomatal lineage like *Arabidopsis* [58]. The basal angiosperms and dicots are basically similar to *A. thaliana* in stomatal development, although many of them show some differences in amplifying divisions [59]. In monocots, the formation of two guard cells needs two steps if not considering the subsidiary cells, i.e., one asymmetric division to generate a GMC, and a symmetric division to form two guard cells [60], [61]. The presence or absence of at least one asymmetric division in the cell lineage leading to the guard mother cell is a potential key factor in land-plant evolution. However, it is unclear which kind of division (symmetric or asymmetric) is ancestral in land plants [55]. More works are needed on the stomatal development of early land plants.

Based on the above information, it is obvious that the basic developmental process of stomata has not greatly changed in land plants if we do not consider the formation of the neighbor cells. At least two steps are necessary and conserved. One is the formation of GMC by one or two symmetric or asymmetric divisions, and the other is the formation of two GCs by a symmetric division. The two steps are directly mediated by the *MUTE* and *FAMA* genes, which has been proved by the experiments that PpaSMF1 and PpaSMF2 can partially rescue the *fama* and *mute* phenotypes [31], and by the fact that the *MUTE* and *FAMA* genes are functionally conserved in the monocots *Oryza sativa* and *Zea mays* [50], and the eudicot *A. thaliana* (Reviewed by [53]).

The *SPCH* gene controls the first asymmetric division of protodermal cells to initiate the stomatal lineage in *Arabidopsis*. However, functions of the two *SPCH* homologs from *Oryza sativa* (*OsaSPCH1*, *OsaSPCH2*) have somewhat diverged. The *OsaSPCH2* plays a role in promoting the early events of the stomatal development whereas *OsaSPCH1* does not [50]. In addition, the *SPCH* genes of angiosperms have experienced more duplication events and harbor more copies than the *MUTE* and *FAMA* genes (Table 1). All this information, together with the variation of the stomatal development in the entry division [33], [58], implies that the *SPCH* genes could have contributed greatly to the diversity of stomatal development in angiosperms.

MacAlister and Bergmann [31] constructed a model to explain the developmental complexity of the stomatal lineage. In their model, a single multifunctional bHLH Ia gene was originally responsible for both specification of GMC identity and GC differentiation in early land plants. Then, the ancestral Ia gene was duplicated into two genes, *MUTE* for GMC identity and *FAMA* for GC differentiation. Finally, a third Ia member, *SPCH*, originated from another gene duplication, which allows for the typical stomatal lineage in angiosperms, including amplifying divisions. This model was based on the phylogeny of the *SPCH/MUTE/FAMA* genes constructed in their study that sampled nine angiosperms, *Picea glauca*, *Physcomitrella patens* and *Selaginella moellendorffii*, and particularly based on their finding that PpaSMF1, PpaSMF2 and SmoSMF1 grouped with the angiosperm MUTEs. However, in that phylogeny, it is difficult to understand that *PglSPCH*, one Ia member from the gymnosperm *P. glauca*, was grouped with the angiosperm *SPCH* genes, although with only 49% bootstrap support, while no *MUTE* gene was found in gymnosperms. Furthermore, that phylogeny is topologically different from the phylogenies constructed in the present and all other previous studies [29], [33], [35], [50]. Therefore, it seems incredible that *MUTE* originated before *SPCH* if the *MUTE* gene does not occur in gymnosperms.

In the present study, all bHLH Ia genes from the two basal land plants *Physcomitrella* and *Selaginella* show close relationships with the *FAMA* genes of gymnosperms and angiosperms, and the angiosperm *SPCH* and *MUTE* genes form a clade sister to the gymnosperm ‘*SPCH*’ genes (Fig. 1). Hence, we agree with MacAlister and Bergmann [31] that an ancestral Ia gene with multifunction could control the specification of GMC identity and GC differentiation in basal land plants. However, we argue that *SPCH* originated earlier than *MUTE* based on the gene phylogeny and structure. The *SPCH* and *MUTE* genes are easily to be identified because they have a MPKTD domain and a truncated N-terminus, respectively. Theoretically, it is easier for *MUTE* to lose the N-terminus than for *SPCH* to gain the MPKTD domain [31]. However, the MPKTD domain could have originated before the divergence of gymnosperms and angiosperms, given the fact that the gymnosperm ‘*SPCH*’ genes have a truncated MPKTD domain whereas the *MUTE* genes are only found in angiosperms. Furthermore, the partial deletion of the MPKTD in *SPCH* generates a protein that more resembles *MUTE* in function [51], also supporting that the *MUTE* gene might have derived from the ancestor of the *SPCH* gene by loss of the MPKTD domain. To reveal the evolutionary history of the MPKTD domain, more species of lycophytes and ferns need to be studied.

**Figure 4:**
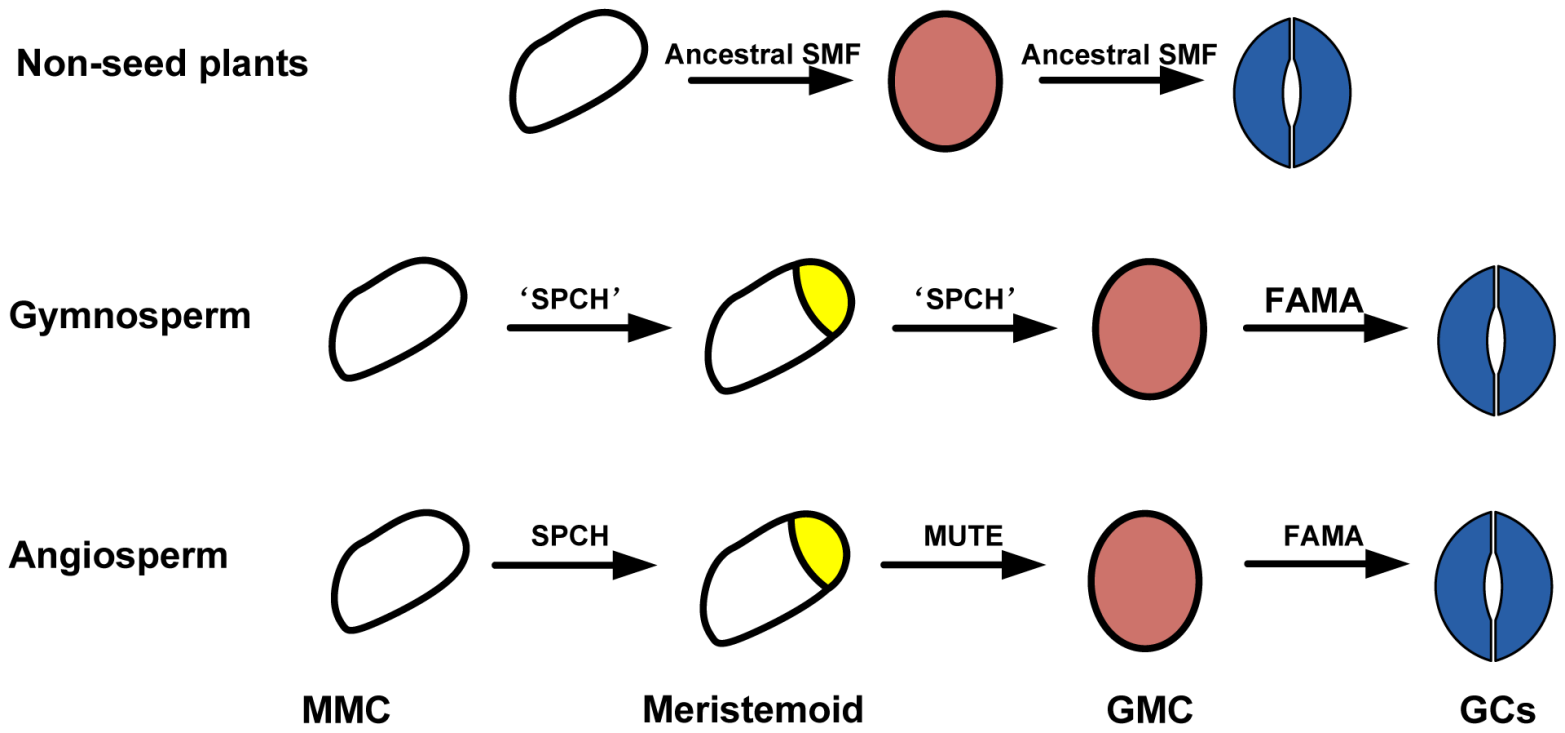
A predictive model of the stomatal development with the evolution of land plants. (A) A single, multifunctional Ia gene responsible for the formation of both guard mother cell (GMC) and guard cells (GCs). (B) An ancestral ‘*SPCH*’ that originally occurred in gymnosperms by a gene duplication has a dual function of *MUTE* and *SPCH* and allows for the divergence between *FAMA* and ‘*SPCH’*. (C) The ‘*SPCH*’ gene evolved into the two genes *SPCH* and *MUTE* by another duplication in the ancestor of angiosperms.

Based on the phylogeny and structure of the bHLH Ia genes (Figs. 1, 2), we deduce that a single multifunctional Ia gene responsible for the stomatal development originated in early land plants. Then, the ancestral Ia gene gave rise to two genes by a duplication in the ancestor of seed plants, one keeping the function of *FAMA* while the other (like the gymnosperm ‘*SPCH*’ genes) acquiring the MPKTD domain and having a dual function of *SPCH* and *MUTE*. Finally, the ‘*SPCH*’ gene evolved into the two genes *SPCH* and *MUTE* by another duplication in the ancestor of angiosperms. The *SPCH* retained the MPKTD domain, whereas the *MUTE* lost it, and got a later start codon at the beginning of the bHLH domain due to the disruption of the initial start codon (Fig. 4). One may argue that the gymnosperm *FAMA* genes could have multifunction as the *FAMA* genes in basal land plants. However, it is clear that the gymnosperm ‘*SPCH*’ genes are sister to the angiosperm *SPCH*-*MUTE* genes (Fig. 1). For a better understanding of functions of the *SPCH/MUTE/FAMA* genes, more functional studies should be done in non-flowering vascular plants in the future.

## Conclusions

In this study, we revealed the evolutionary relationships of three bHLH Ia genes (*SPCH*, *MUTE* and *FAMA*) of land plants that mediate sequential steps of stomatal development based on a wide sampling. The most interesting findings include the close relationship between the Ia genes from basal land plants (*Physcomitrella* and *Selaginella*) and the *FAMA* genes of seed plants, and the sister relationship between the gymnosperm ‘*SPCH*’ genes and the angiosperm *SPCH* and *MUTE* genes. Also, we proposed a model for the correlation between the evolution of stomatal development and the genes involved in this developmental process in land plants. However, only a few of bHLH Ia genes of gymnosperms that are available were included in this study, and almost nothing is known about the functions of these genes. It would be of great interest to investigate evolution and function of the ‘*SPCH*’ and *FAMA* genes in more gymnosperms in the future.

## Supporting Information

Figure S1 **Phylogeny of the bHLH Ia genes with a reconstruction of ancestral gene structure.**
(PDF)

Figure S2 **Maximum-likelihood (ML) trees of the bHLH Ia genes.** A, A rooted ML tree based on amino acid sequences with the bHLH Ia genes from *Physcomitrella patens* as outgroups. B, A rooted tree based on nucleotide sequences with the bHLH Ia genes from *P. patens* as outgroups. C, An unrooted ML tree of the Ia genes constructed based on amino acid sequences. Numbers above branches refer to bootstrap values higher than 50%. The stars denote some inferred duplication events. Gene names are shown in Table S1. Ang, angiosperms; Gym, gymnosperms; Smo, *moellendorffii*; Ppa, *Physcomitrella patens*.
(PDF)

Table S1 **The sources of the Ia members of the bHLH transcriptional factors we analyzed.**
(XLS)

Table S2 **The primers used in this study.** * represent the primers used in the gene amplification.
(DOC)

Table S3 **Results of the site and branch-site analyses of the** ***SPCH/MUTE/SPCH*** **genes.**
(XLS)
