# Supplementary figures and images for: Evolution of the bHLH Genes Involved in Stomatal Development: Implications for the Expansion of Developmental Complexity of Stomata in Land Plants
Source: PLoS One. 2013 Nov 11;8(11):e78997. doi: 10.1371/journal.pone.0078997 (PMC3823973; doi:10.1371/journal.pone.0078997)

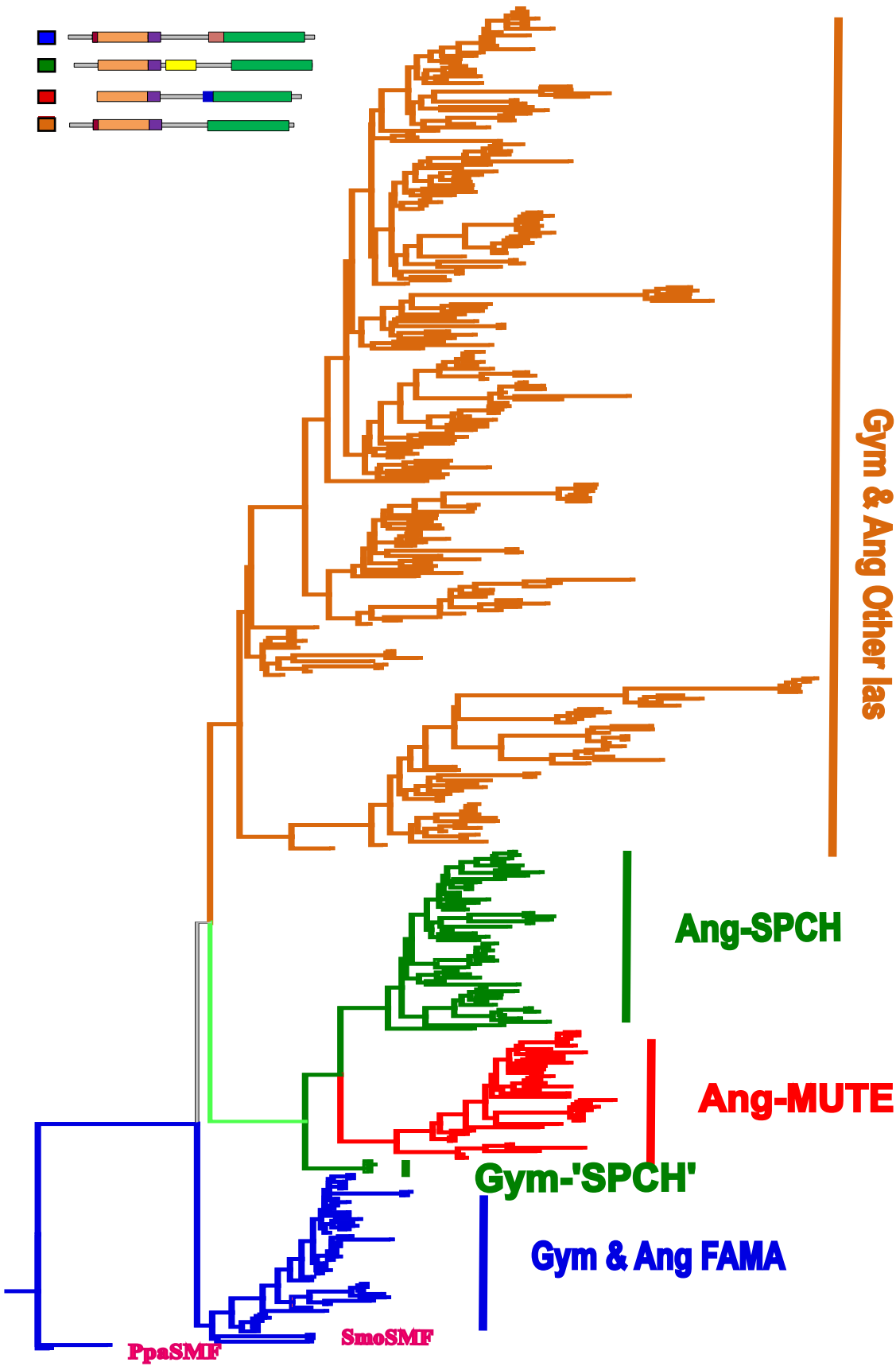

Supplement: Figure S1 — Phylogeny of the bHLH Ia genes with a reconstruction of ancestral gene structure. (PDF) [file pone.0078997.s001.pdf]

## Gym & Ang other las

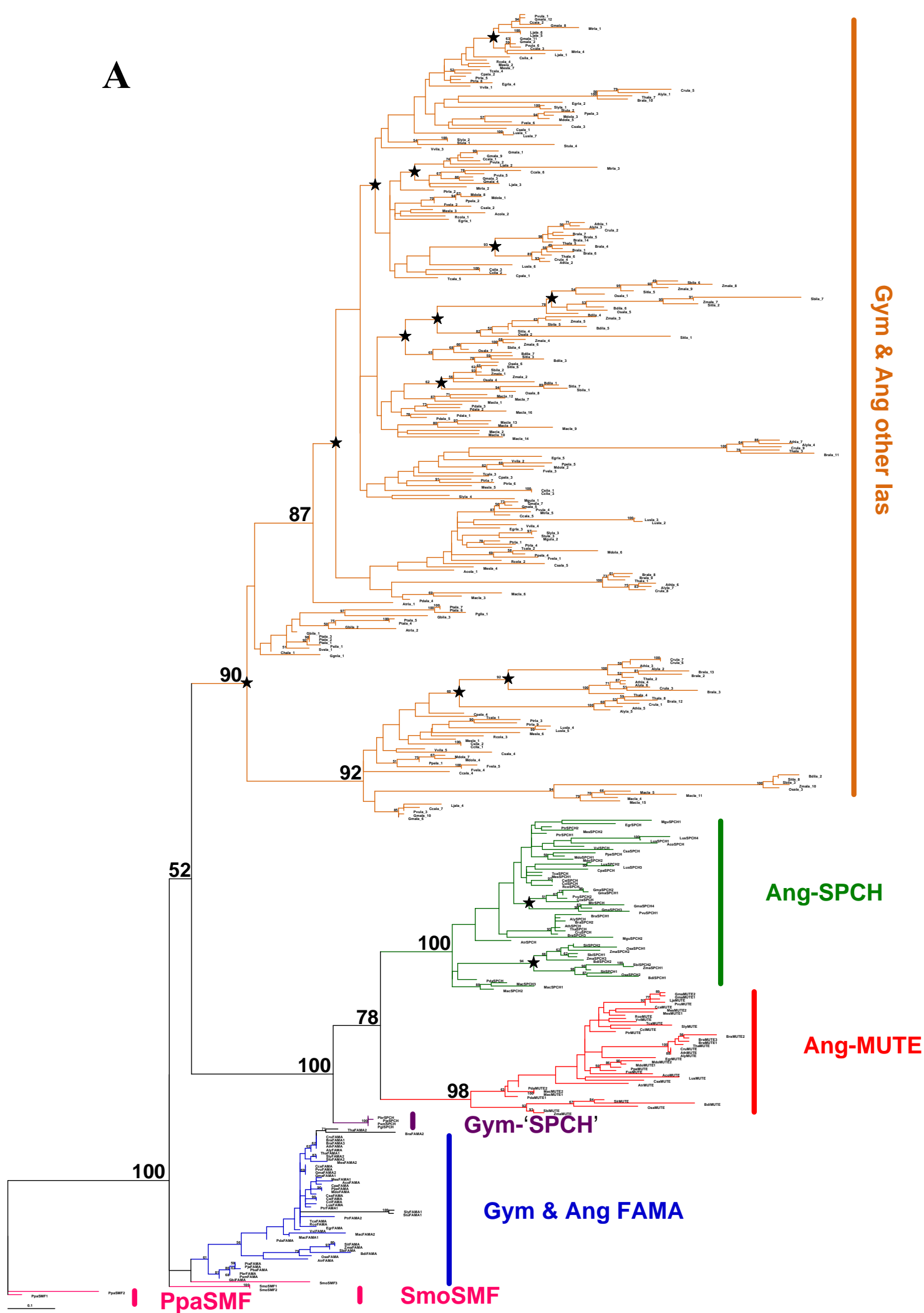

B

Gym & Ang other las

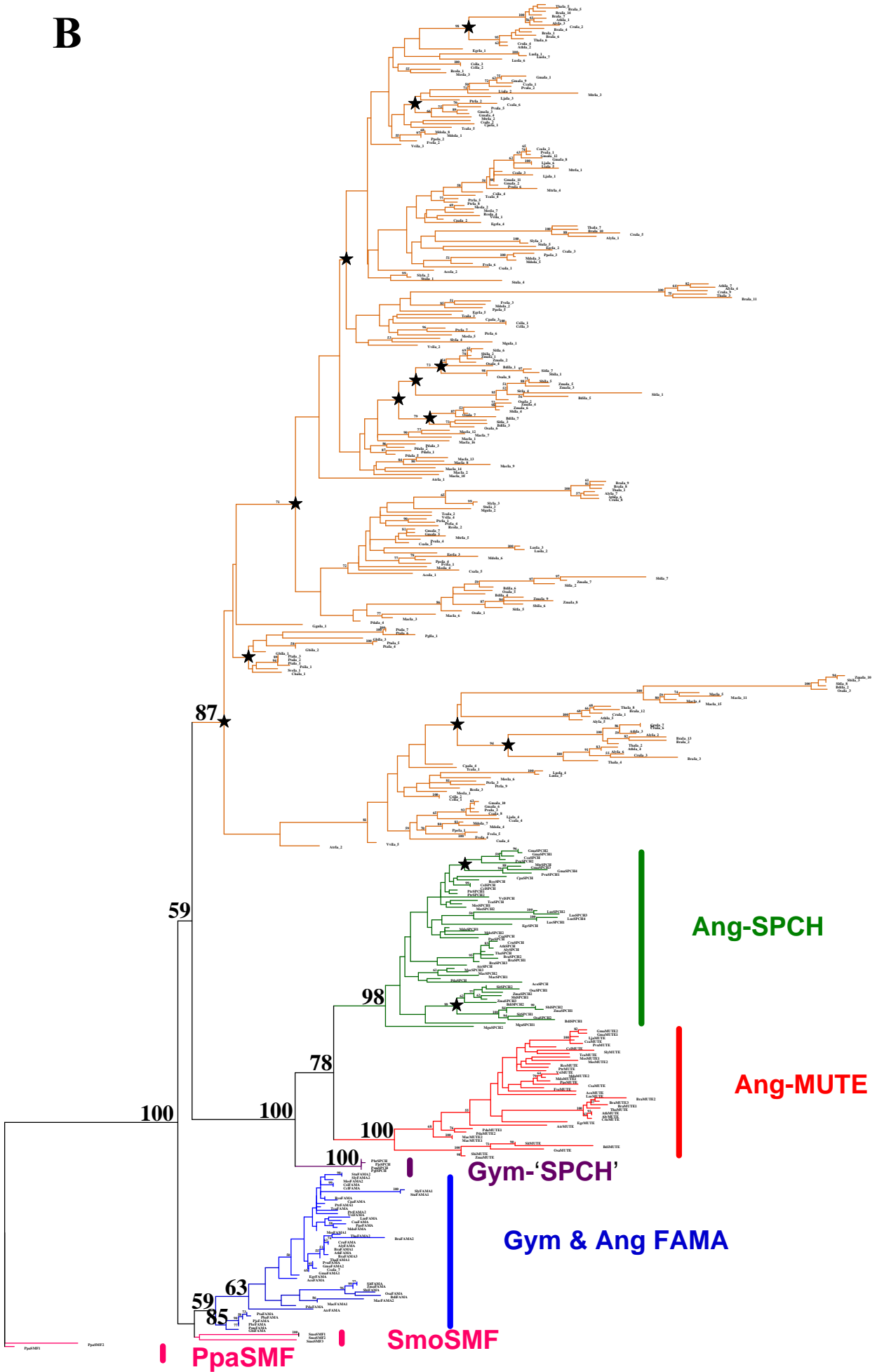

C

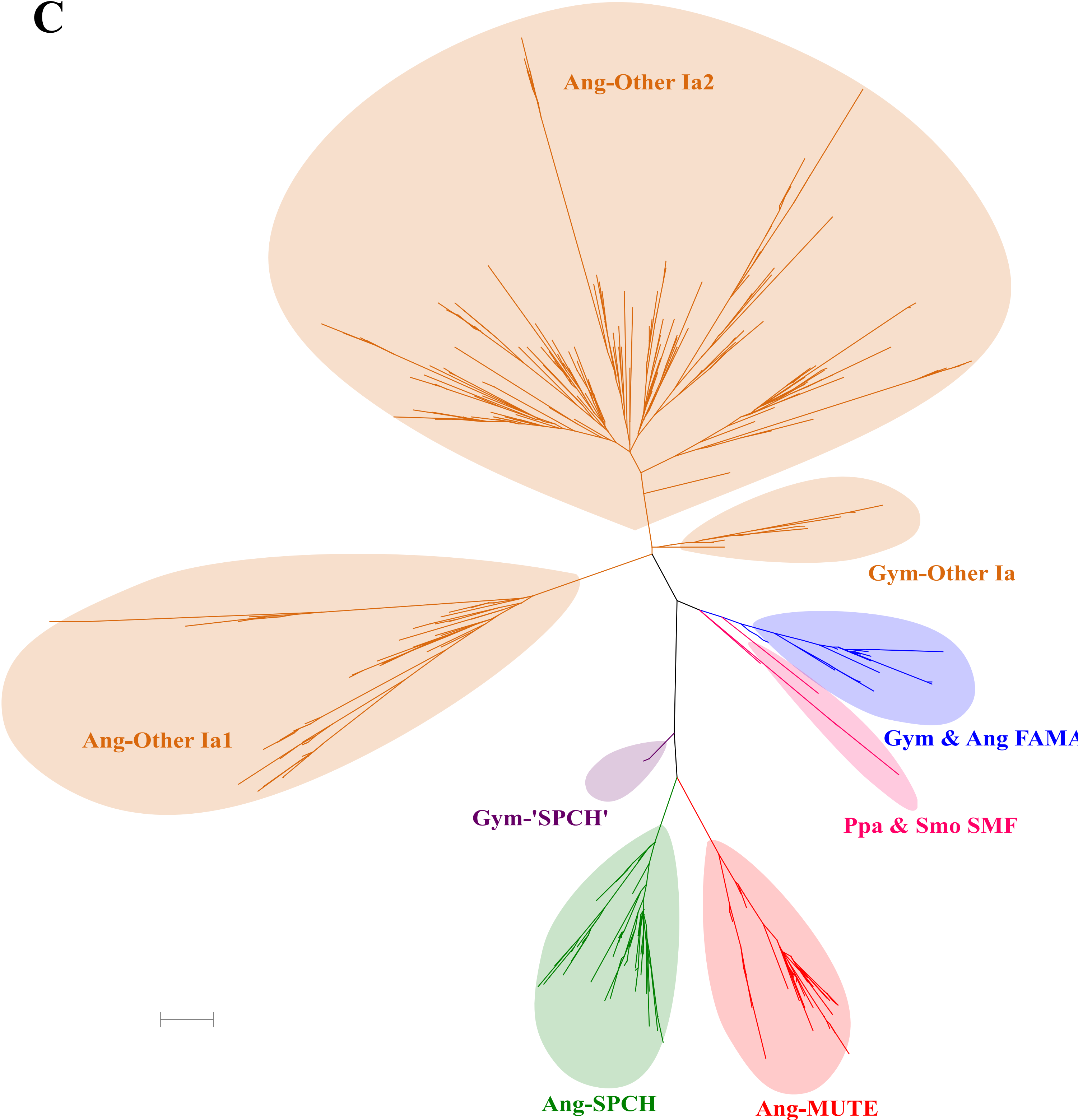

Supplement: Figure S2 — Maximum-likelihood (ML) trees of the bHLH Ia genes. A, A rooted ML tree based on amino acid sequences with the bHLH Ia genes from Physcomitrella patens as outgroups. B, A rooted tree based on nucleotide sequences with the bHLH Ia genes from P. patens as outgroups. C, An unrooted ML tree of the Ia genes constructed based on amino acid sequences. Numbers above branches refer to bootstrap values higher than 50%. The stars denote some inferred duplication events. Gene names are shown in Table S1. Ang, angiosperms; Gym, gymnosperms; Smo, moellendorffii; Ppa, Physcomitrella patens. (PDF) [file pone.0078997.s002.pdf]
