# Supplementary material for: Evolution of the bHLH Genes Involved in Stomatal Development: Implications for the Expansion of Developmental Complexity of Stomata in Land Plants
Source: PLoS One. 2013 Nov 11;8(11):e78997. doi: 10.1371/journal.pone.0078997 (PMC3823973; doi:10.1371/journal.pone.0078997)
Supplement: Table S2 — The primers used in this study. * represent the primers used in the gene amplification. (DOC) [file pone.0078997.s004.doc]

**Table S2. The primers used in this study.** * represent the primers used in the gene amplification.

| **Primers** | **Sequences (5’-3’)** |
| --- | --- |
| FAMA-U5F3* | AGAACAGTGAAGAAGTYGAAAGCC |
| FAMA-U3R* | TGCTGTTCCTCTTCATTGCCAC |
| bHLH2-U5F* | AACAATAAAGGGAATATGGTCGG |
| bHLH2-U3R1* | CCTTCACTGGAGGGTTAGCAT |
| FAMA-W-2 | TCTTTAGGAGCTGGCCTGGTCTC |
| FAMA-W-4 | TGCGAT TCTAAG CATTGCAGG AG |
| bHLH2-F2D | AGGCATCAA TCATAGGCGGAGTT |
| bHLH2-R1 | ACTCCGCCTATGATTGATGC |
| bHLH2-seqR | TGAGAACCGTGTCGTCTATTGTG |
